# Using country-level variables to classify countries according to the number of confirmed COVID-19 cases: An unsupervised machine learning approach

**DOI:** 10.12688/wellcomeopenres.15819.3

**Published:** 2020-06-15

**Authors:** Rodrigo M. Carrillo-Larco, Manuel Castillo-Cara

**Affiliations:** 1 Department of Epidemiology and Biostatistics, School of Public Health, Imperial College London, London, UK; 2 CRONICAS Centre of Excellence in Chronic Diseases, Universidad Peruana Cayetano Heredia, Lima, Peru; 3 Universidad Católica de Trullijo, Instituto de Investigación, Chimbote, Peru; 4 Center of Information and Communication Technologies, Universidad Nacional de Ingeniería, Lima, Peru

**Keywords:** COVID-19, pandemic, clustering, k-mean, unsupervised algorithms

## Abstract

**Background: **The COVID-19 pandemic has attracted the attention of researchers and clinicians whom have provided evidence about risk factors and clinical outcomes. Research on the COVID-19 pandemic benefiting from open-access data and machine learning algorithms is still scarce yet can produce relevant and pragmatic information. With country-level pre-COVID-19-pandemic variables, we aimed to cluster countries in groups with shared profiles of the COVID-19 pandemic.

**Methods: **Unsupervised machine learning algorithms (k-means) were used to define data-driven clusters of countries; the algorithm was informed by disease prevalence estimates, metrics of air pollution, socio-economic status and health system coverage. Using the one-way ANOVA test, we compared the clusters in terms of number of confirmed COVID-19 cases, number of deaths, case fatality rate and order in which the country reported the first case.

**Results: **The model to define the clusters was developed with 155 countries. The model with three principal component analysis parameters and five or six clusters showed the best ability to group countries in relevant sets. There was strong evidence that the model with five or six clusters could stratify countries according to the number of confirmed COVID-19 cases (p<0.001). However, the model could not stratify countries in terms of number of deaths or case fatality rate.

**Conclusions**: A simple data-driven approach using available global information before the COVID-19 pandemic, seemed able to classify countries in terms of the number of confirmed COVID-19 cases. The model was not able to stratify countries based on COVID-19 mortality data.

## Introduction

The ongoing COVID-19 pandemic has attracted the attention and interest of public health officers, practitioners, researchers and the general population. They all are working together to slow down the spread of the disease, thus reducing the number of severe cases and deaths. Their efforts have already produced relevant preliminary information on COVID-19 risk factors and the epidemiological profile of the disease ^1– 3^, with plenty more information not published yet (e.g., academic pre-prints).

The available evidence—published and unpublished—has mostly focused on the individual level; that is, they have studied the patients, their characteristics, disease progression and outcomes. Little has been studied about large populations and geographic areas; in other words, ecological evidence and research addressing study units other than the patients are scarce, though can reveal relevant and pragmatic information. In this line, research with novel analytical approaches, such as machine learning algorithms, is also uncommon.

Research at the country level could reveal potentially modifiable associated factors that individual-level data are still unable to study because of the limited number of observations. Moreover, machine learning techniques informed by country-level variables can provide classification algorithms useful to understand how countries may behave during and after the COVID-19 pandemic. Therefore, classification algorithms can reveal patterns to identify countries where the pandemic may have a similar effect. Countries could use this information to prevent worse-case scenarios given the cluster to which they belong. Global and regional organizations could use country clusters to organize similar aid to countries in the same cluster, while prioritizing clusters likely to experience the worse outcomes. Consequently, we aimed to develop a simple unsupervised machine learning algorithm informed by country-level variables before the COVID-19 pandemic, that can classify countries regarding the number of confirmed COVID-19 cases and deaths. That is, we aimed to answer: *can country characteristics before the COVID-19 pandemic be useful to cluster countries according to COVID-19 outcomes (e.g., number of cases and deaths)?* In so doing, we provide a preliminary framework to stratify countries with similar progression through the COVID-19 pandemic.

## Methods

### Data sources

We used different data sources to build a dataset with information on COVID-19, prevalence estimates of selected diseases, a socio-economic metric, an air pollution metric, and a metric of health system coverage ( Table 1). The unit of analysis was a country. Variables and specific data sources are shown in Table 1. Except for the COVID-19 variables, the other variables were used in the clustering analysis; that is, we used eight input variables for the cluster analysis: four diseases, air quality, gross domestic product per-capita, an universal health coverage index and the proportion of men in the country ( Table 1). In other words, countries were clustered following unsupervised machine learning algorithms based on prevalence estimates of the selected diseases, socio-economic status, air pollution and health system coverage ( Table 1).

**Table 1.** Extracted data, variables and data sources.

Concept	Variables	Data source	Used for
COVID-19 prevalence	Country; number of confirmed cases (as of 23/03/2020); number of confirmed deaths (as of 23/03/2020); case fatality rate per 1,000 cases (as of 23/03/2020); order number at which the country experienced the first case (e.g., 1 ^st^ country, 2 ^nd^ country…)	COVID-19 global surveillance system by Johns Hopkins University ^4, 5^	Cluster evaluation
Disease prevalence	Age-standardized prevalence of diabetes, chronic obstructive disease [COPD], HIV/AIDS and tuberculosis (as of year 2017)	2017 Global Burden of Disease / Institute for Health Metrics, Washington University ^6^	Clustering
Male population	Proportion of males in the country		
Air quality metric	Concentration of 2.5 particulate matter by country	Global Health Observatory data repository, World Health Organization ^7^	Clustering
Socio-economic metric	Gross domestic product per capita (as of year 2017) ^a^	World Bank ^8^	Clustering
Health system metric	Universal health coverage index of service coverage (as of year 2017)	Global Health Observatory data repository, World Health Organization ^9^	Clustering

^a^When a country did not have data for 2017, we used the latest available; when a country did not have any data on this source, we used data as reported by a Google search (this was the case for four countries).

These predictors were selected because they are closely related to the COVID-19 pandemic, both from a clinical and public health perspective. We chose two chronic non-communicable diseases (diabetes and chronic obstructive pulmonary disease [COPD]) and two infectious diseases (tuberculosis and HIV/IDS). Diabetes seems to be very frequent among COVID-19 patients ^10^. Although hypertension had a higher frequency than respiratory diseases ^10^, we chose COPD because of the structural and pathophysiological pathways it can share with an acute respiratory disease such as COVID-19; the same logic would apply for tuberculosis. We chose HIV/AIDS because of the high potential of impaired immune response. We chose 2.5 particulate matter (particles of width <2.5 µm) as a metric of air pollution; 2.5 particulate matter has been related to severe acute respiratory syndrome ^11^. Finally, we chose a metric of socio-economic status and health system coverage, which could impact on the probability of a person to adopt preventive care and access to appropriate healthcare should it be necessary.

### Data analysis – clustering

*Predictors.* The variables used to develop the clustering model had different values between them, thus each of them carries a different variance. Because of this characteristic, it is relevant to standardize these variables to set reliable clusters without losing information. Consequently, before running the unsupervised clustering algorithms, the predictors were treated with an orthogonal transformation and then with principal component analysis (PCA).

*PCA.* The PCA is a technique within the remit of unsupervised machine learning algorithms. PCA follows an orthogonal transformation, which turns correlated variables into an uncorrelated set of variables. The PCA aims to create a set of characteristics, or components, that represents the relevant information from the original group of variables ^12, 13^. The PCA seeks to reduce the number of predictors while maximizing the variance.

In this work, and to avoid losing information explained by the original eight predictors, we prespecified three PCA components; the three PCA components retained a variance of 1. This method of obtaining 100% as an explained variance imply keeping 100% of the information explained by the original eight predictors. Moreover, these three components gave the most reliable clusters as reported in the results section. We used the PCA algorithm available in the Scikit-Learn library ^14^.

*K-means.* This technique seeks to group heterogenous elements into homogenous clusters. This approach is considered a paradigm in unsupervised machine learning, because it assigns the elements into clusters which were unknown at the beginning of the analysis ^15^. A few authors have used this methodology in clinical and public health research ^16– 19^.

There are different methods for unsupervised clustering depending on the data characteristics ^20^. Given our data and aims, we chose a centroid-based algorithm: k-means. This approach works well when the clusters have similar size, similar densities and follow a globular shape.

**Figure 1.**
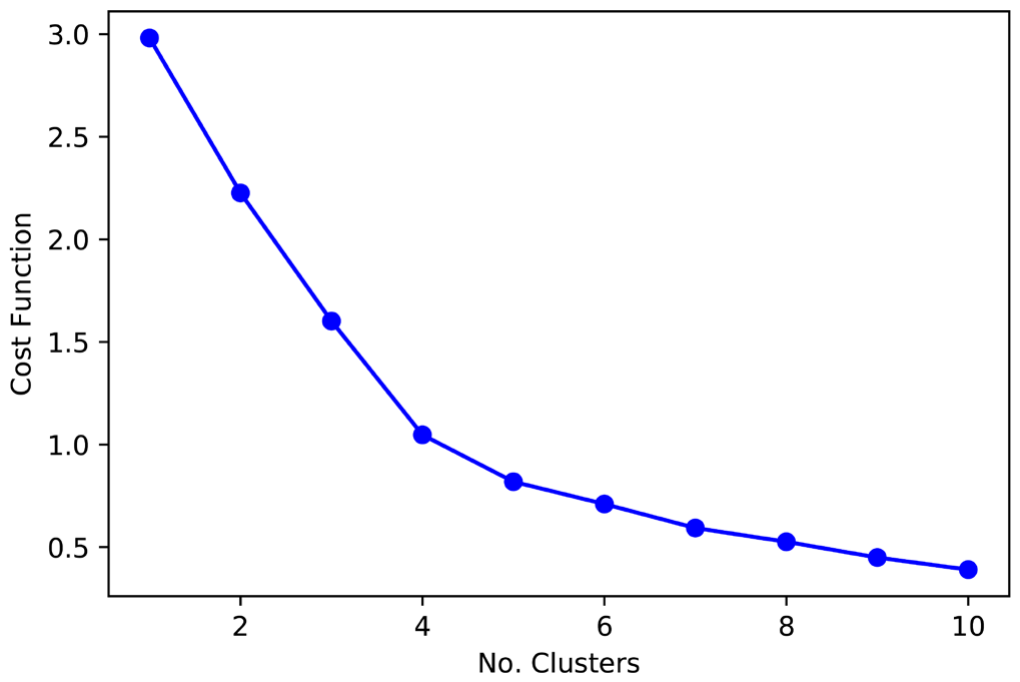
Cost function for the k-mean analysis.

*Number of clusters*. Regarding the number of clusters that optimizes the function convergence to the centroids, we plotted the elbow function ( Figure 1) which, paired with epidemiological knowledge from the countries, supported the choose of five and six clusters ( Figure 1). That is, five and six cluster classified countries in groups with shared socio-demographic and epidemiological profiles. Although five and six clusters provided similar groups, six clusters classified central Africa with greater detail, which could be useful for these countries and regional organizations. Overall, the function cost (elbow plot, Figure 1), paired with the overall results (boxplots and maps), suggested that five or six cluster were a sensitive decision.

When there is a limited number of observations, as it is arguably in this analysis, the number of clusters around the “elbow” function ( Figure 1) provides similar information. At this point, it may be advisable to select the number of clusters which relates better to expert knowledge. Therefore, we used visual inspection of maps and plots to decide on the number of clusters that provide the best results, grouping countries in consistent clusters with similar background.

Post-hoc analysis suggested we made a sensible choice when selecting 5 and 6 clusters. A dendrogram with Euclidean distances showed that 5 clusters were the optimum number. Similarly, the Silhouette analysis revealed the largest average Silhouette score for 3 (0.43), 4 (0.48), 5 (0.44), and 6 (0.42) clusters; all other options from 1 to 10 clusters were below 0.40.

As explained above, the visual inspection of maps suggested that 3 or 4 clusters did not provide a good classification. That is, countries with no strong similarities were clustered. Visual inspection of the maps was based on geopolitical, geographical and epidemiological knowledge, in general and regarding the input variables. A segmentation in 4 cluster did not reveal interesting, reliable or expected groups; in other words, based on background knowledge, countries expected to be together were not. A segmentation in 5 and 6 clusters provided sensible results in accordance with prior knowledge. Overall, our choice of 5 and 6 clusters was sensible, based on prior knowledge and still supported by the analysed metrics (dendrogram and Silhouette).

We used the k-mean algorithm available in the Scikit-Learn library, with five and six clusters, 500 iterations, and a fast initiation of convergence with k-mean++ ^21^.

### Statistical analysis

The COVID-19 variables—number of confirmed cases, number of deaths, case fatality rate and order when the first case appeared—were compared across clusters with the one-way ANOVA tests. Within clusters, pairwise combinations were analysed with t-tests adjusted for multiple comparisons with the Bonferroni method. The statistical analysis was conducted with COVID-19 data until March 23 ^rd^, 2020. Analysis was performed in R (v3.6.1).

### Ethics

This work analysed open-access data and did not involve any human subjects. No approval by an IRB or ethics committee was sought.

## Results

### Data points

**Figure 2.**
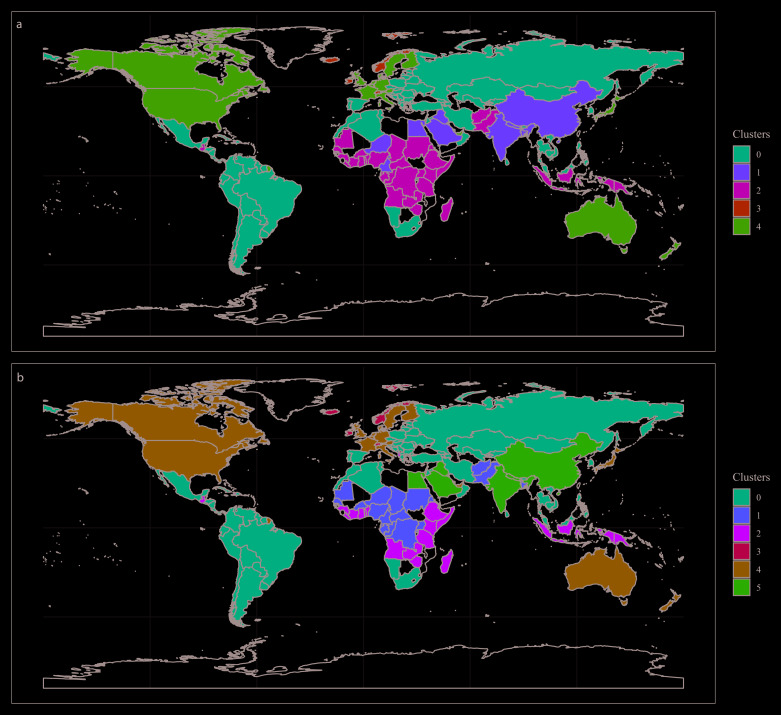
World map showing countries coloured as per the model with five ( **A**) and six ( **B**) clusters.

**Figure 3.**
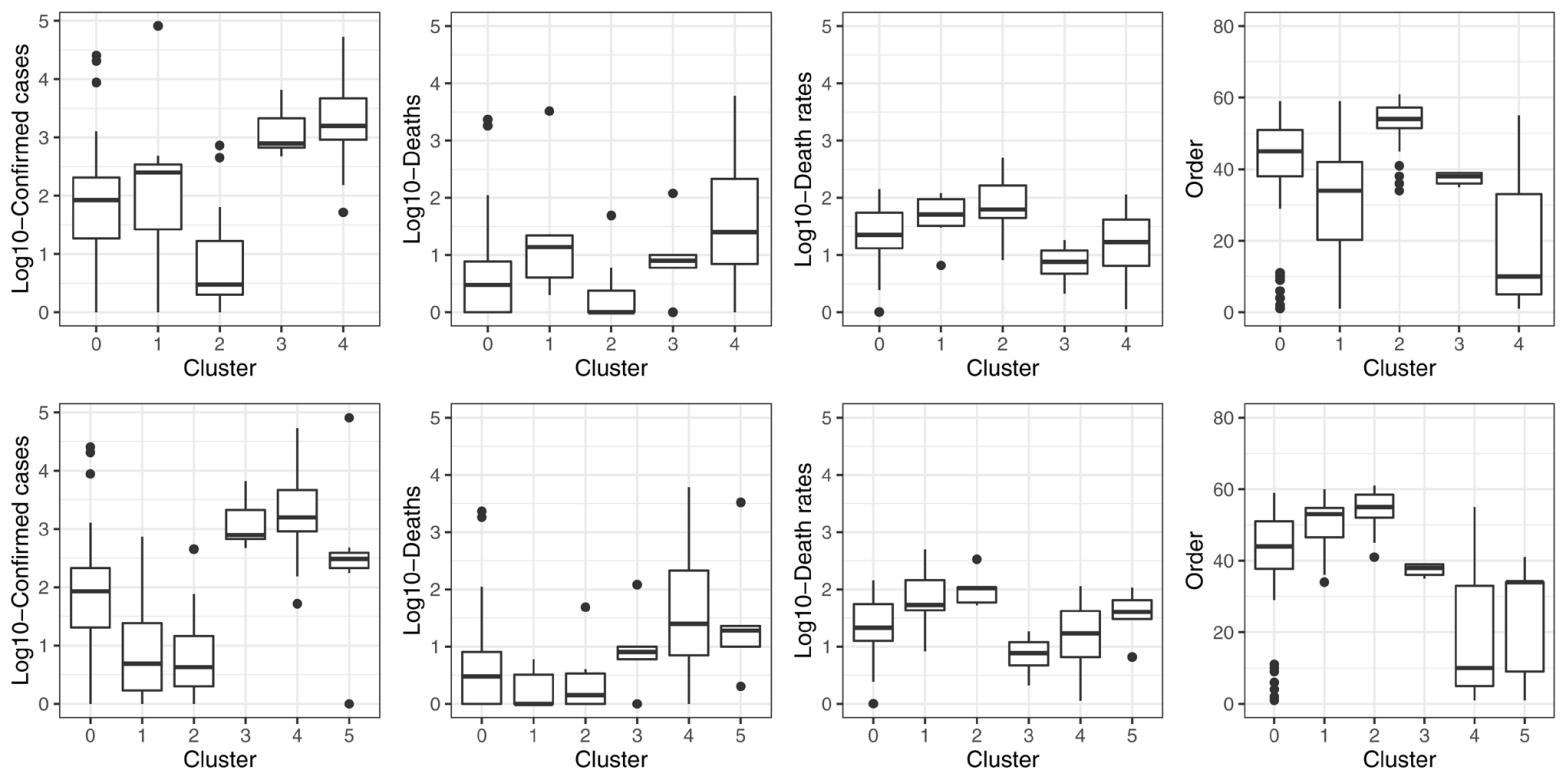
Boxplots showing the distribution of COVID-19 pandemic variables across clusters.

The clustering models were built with 155 countries and territories. Based on visual inspection of maps and boxplots, and on statistical parameters, the clustering models with three PCA components and five ( Figure 2A) or six ( Figure 2B) clusters performed the best to stratify countries according to COVID-19 variables ( Figure 3; data available with the manuscript). The median and interquartile range, of the variables used in the clustering analysis, are presented in Table 2.

**Table 2.** Characteristics of the input variables across clusters.

Cluster #	5 clusters			6 clusters		
	1st quartile	Median	3rd quartile	1st quartile	Median	3rd quartile
	Diabetes prevalence (%)					
0	6.10	7.33	8.93	6.13	7.38	8.96
1	5.41	7.16	9.75	5.48	6.47	9.28
2	5.79	6.50	8.88	5.69	6.38	8.56
3	7.38	7.78	8.70	7.38	7.78	8.70
4	5.56	6.65	7.66	5.56	6.65	7.66
5				5.69	9.19	10.03
	Chronic pulmonary obstructive disease prevalence (%)					
0	2.75	3.64	4.21	2.78	3.64	4.21
1	3.44	3.77	4.02	2.44	3.26	3.47
2	2.44	3.21	3.46	2.78	3.23	3.61
3	4.05	4.41	4.45	4.05	4.41	4.45
4	3.12	3.54	4.54	3.12	3.54	4.54
5				3.36	3.80	4.47
	HIV/AIDS prevalence (%)					
0	0.03	0.11	0.34	0.03	0.12	0.34
1	0.01	0.02	0.14	0.30	0.88	2.41
2	0.40	1.14	2.30	0.23	1.06	1.68
3	0.09	0.11	0.12	0.09	0.11	0.12
4	0.07	0.12	0.17	0.07	0.13	0.17
5				0.01	0.02	0.04
	Tuberculosis prevalence (%)					
0	12.58	17.51	22.02	12.49	17.29	21.94
1	14.45	22.78	28.76	23.77	28.98	34.10
2	27.16	31.57	35.96	28.32	31.90	36.44
3	7.09	7.21	7.33	7.09	7.21	7.33
4	7.52	8.45	10.55	7.52	8.45	10.55
5				14.79	22.48	24.01
	Concentration of 2.5 particulate matter					
0	15.00	18.40	24.15	15.05	18.40	24.07
1	58.15	67.15	78.62	39.98	46.90	53.05
2	23.688	32.90	41.20	17.90	23.65	20.10
3	7.00	8.30	10.20	7.00	8.30	10.20
4	7.30	11.60	14.10	7.30	11.60	14.10
5				57.70	69.00	79.30
	Gross domestic product per capita					
0	4,155	7,609	15,083	4,159	7,697	15,139
1	1,528	3,822	21,531	619	1,256	1,769
2	658	1,256	2,006	766	1,546	2,527
3	71,315	75,497	80,450	71,315	75,497	80,450
4	40,087	44,240	51,150	40,087	44,240	51,150
5				2,440	8,759	23,715
	Universal health coverage index of service coverage					
0	68.0	73.0	76.0	69.0	73.0	76.0
1	48.0	64.5	74.5	38.8	43.0	45.3
2	39.0	43.0	47.0	40.0	45.5	53.8
3	83.0	83.0	84.0	83.0	83.0	84.0
4	80.0	83.0	86.0	81.0	83.0	86.0
5				61.0	68.0	76.0
	Male proportion (%)					
0	48.56	49.43	50.20	48.56	49.42	50.17
1	49.77	51.37	55.27	49.18	49.71	50.20
2	48.64	49.71	50.49	48.62	49.68	50.44
3	49.75	50.24	50.38	49.75	50.24	50.38
4	48.73	49.22	49.51	48.73	49.22	49.51
5				51.30	51.59	58.10

### Clusters prediction

The one-way ANOVA test comparing the confirmed number of COVID-19 cases across the five and six clusters, strongly suggested there was a difference between groups (p<0.001). Regarding the model with five clusters, the strongest differences were between clusters 0 and 1, 0 and 4, 1 and 2, 2 and 3, as well as 2 and 4 ( Figure 3, Table 3). Similarly, for the model with six clusters there were ten pairwise combinations with strong differences in the number of confirmed COVID-19 cases ( Figure 3, Table 3).

**Table 3.** Pairwise combinations between clusters according to COVID-19 variables (as of March 23 ^rd^, 2020).

	Number of confirmed cases					Number of confirmed cases				
**Clusters**	**0**	**1**	**2**	**3**	**Clusters**	**0**	**1**	**2**	**3**	**4**
**1**	1.000				**1**	<0.001				
**2**	<0.001	<0.001			**2**	<0.001	1.000			
**3**	0.023	0.300	<0.001		**3**	0.034	<0.001	<0.001		
**4**	<0.001	0.003	<0.001	1.000	**4**	<0.001	<0.001	<0.001	1.000	
					**5**	0.771	<0.001	<0.001	1.000	0.270
	Number of deaths					Number of deaths				
**Clusters**	**0**	**1**	**2**	**3**	**Clusters**	**0**	**1**	**2**	**3**	**4**
**1**	1.000				**1**	1.000				
**2**	1.000	1.000			**2**	1.000	1.000			
**3**	1.000	1.000	1.000		**3**	1.000	1.000	1.000		
**4**	0.110	1.000	0.096	1.000	**4**	0.180	0.320	0.290	1.000	
					**5**	1.000	1.000	1.000	1.000	1.000
	**Case fatality rate per 1,000 cases**					**Case fatality rate per 1,000 cases**				
**Clusters**	**0**	**1**	**2**	**3**	**Clusters**	**0**	**1**	**2**	**3**	**4**
**1**	1.000				**1**	0.460				
**2**	0.430	1.000			**2**	1.000	1.000			
**3**	1.000	1.000	1.000		**3**	1.000	1.000	1.000		
**4**	1.000	1.000	1.000	1.000	**4**	1.000	1.000	1.000	1.000	
					**5**	1.000	1.000	1.000	1.000	1.000
	**Order**					**Order**				
**Clusters**	**0**	**1**	**2**	**3**	**Clusters**	**0**	**1**	**2**	**3**	**4**
**1**	0.123				**1**	0.064				
**2**	<0.001	<0.001			**2**	<0.002	1.000			
**3**	1.000	1.000	0.198		**3**	1.000	0.649	0.169		
**4**	<0.001	0.040	<0.001	0.025	**4**	<0.001	<0.001	<0.001	0.007	
					**5**	0.004	<0.001	<0.001	1.000	0.856

Cells in red show not significant results (p>0.05); cells in yellow show significant results (p<0.05 & p>0.001); cells in green show strong significant results (p<0.001).

The proposed clustering with five groups did not stratify well according to number of total deaths (p=0.067); adding one more cluster did not improve the prediction (p=0.864). None of the pairwise combinations revealed a strong difference ( Figure 3, Table 3). Overall, the same findings applied to case fatality rate for five (p=0.320) and six (p=0.373) clusters, with no differences in pairwise comparisons ( Figure 3, Table 3).

There was strong difference among cluster regarding the order at which each country had the first confirmed case, regardless of the number of clusters (p<0.001). For the model with five clusters, there were strong pairwise differences in all but four pairs ( Figure 3, Table 3). In a similar line, eight of the pairwise combinations in the model with six clusters revealed a strong difference ( Figure 3, Table 3)

## Discussion

### Main results

Based on open-access variables at the country level, along with unsupervised machine learning algorithms (k-means), we developed a clustering model that can classify countries well regarding the number of confirmed COVID-19 cases. However, the model did not stratify countries well according to the number of deaths or case fatality rate.

The clustering model we proposed has potential applications. First, for each cluster we report a median and a range of number of confirmed COVID-19 cases. Although still early and deserving of further scrutiny as the outbreak progresses, the results could suggest that the number of cases in one country in one cluster will be within the proposed range for that cluster, unless one country performs below the expectation (i.e., exceeds the proposed range).

Unless there are substantial changes in the predictors used to define the clusters, these could signal countries that are particularly vulnerable or resilient for future respiratory outbreaks of this kind. Future research in a similar situation can test whether the proposed clusters also stratify countries well regarding the number of cases. Alternatively, the model could be tested with data of old respiratory pandemics to assess if it would have classified countries well.

Overall, considering the limitations of this work, the stage of the ongoing COVID-19 pandemic, and the general knowledge about this disease and its epidemiological profile, we provided a preliminary clustering model that could be useful to understand similarities and differences across countries, and how they may be affected by the ongoing pandemic.

### Results in context

The input variables could potentially explain the clusters configuration. For example, cluster number four had the largest number of confirmed cases. This cluster also had the best universal health coverage index. It could be argued that such a strong health system is capable of performing tests to large populations, hence a large number of diagnosed cases. Conversely, cluster number two appeared to have the worst death rates; this cluster also had the largest tuberculosis prevalence as well as the smallest gross domestic product per capita and universal health coverage index. These epidemiological –large burden tuberculosis – and socio-demographic profiles could explain why the high death rates.

The cluster configuration herein presented did not seem to group countries closer to China, where the pandemic started. In other words, countries with the first imported cases did not cluster together. This could mean that the selected input variables do not correlate well with, for example, travel frequency or population movement from China to nearby countries. Alternatively, this unexpected finding could suggest that the selected input variables are more relevant than proximity or connections between countries.

We are unaware of other studies that have aimed to classify countries based on simple open-access variables, and that can stratify the countries based on the number of COVID-19 cases. Most of the previous research using unsupervised machine learning clustering algorithms on health research has focused on individuals and diseases ^16– 19^. This work complements the available evidence at the individual level with preliminary information on clusters at the country level, with potential relevant applications in the current COVID-19 pandemic. Nevertheless, future research should verify the accuracy and stability of our findings, so that they can be applied for this and future similar scenarios.

### Strengths and limitations

We proposed a simple algorithm to classify countries regarding the number of confirmed COVID-19 cases. In that sense, this model and others can be easily applied and developed. However, there are limitations to acknowledge. First, one could argue that there were few predictors to define the clusters. However, these were relevant variables that are freely available for research and analysis. Moreover, finding reliable, consistent and comparable information for all -or most- countries in the world may be challenging. This calls to researchers and international organizations to produce more information at the country level following similar methods that will allow global comparisons and analysis. Second, we did not find any strong evidence for the total number of deaths or case fatality rate. This could be because there are, fortunately, still very few deaths in most countries precluding strong comparisons. Our model can be tested again in the future, when the outbreak ends and there would be potentially more deaths, to assess whether the performance on this outcome improves. Third, we based our analysis on the confirmed number of cases and deaths. It is expected that this number may not reflect the actual number of people with the disease. In other words, it is more likely that there are more COVID-19 cases that have not been diagnosed or confirmed. This could be a limitation if we had aimed to predict the exact number of sick people, in which case we should have somehow accounted for the under-reporting.

## Conclusions

Using readily available variables we developed an unsupervised machine learning algorithm that can stratify countries based on the number of COVID-19 confirmed and reported cases. This preliminary work provides a timely algorithm that could help identify countries more vulnerary or resistant to the ongoing pandemic.

## Data availability

### Source data

The source data for this study are described in Table 1.

### Extended data

Figshare: Using country-level variables to classify countries according to the number of confirmed COVID-19 cases: An unsupervised machine learning approach. https://doi.org/10.6084/m9.figshare.12030363.v1 ^22^.

This project contains the following extended data:

- Datasets.zip (containing the pooled data used in this analysis).
- Codes.zip (containing codes used in the analysis to develop the cluster and to assess its performance).

Extended data are available under the terms of the Creative Commons Attribution 4.0 International license (CC-BY 4.0).
