# Supplementary material for: Using country-level variables to classify countries according to the number of confirmed COVID-19 cases: An unsupervised machine learning approach
Source: Wellcome Open Res. 2020 Jun 15;5:56. Originally published 2020 Mar 31. [Version 3] doi: 10.12688/wellcomeopenres.15819.3 (PMC7308996; doi:10.12688/wellcomeopenres.15819.3)
Supplement: Peer review history

# Peer review: Reviewer response for version 3

**DOI:** 10.21956/wellcomeopenres.17626.r39115

**Published:** 2020-06-16

**Authors:** Maria Pikoula, Nonie Alexander

**Affiliations:** 1 Institute of Health Informatics, University College London, London, UK

The authors have meticulously responded to all our comments. We are happy with the latest version of the manuscript.

Is the work clearly and accurately presented and does it cite the current literature?

Yes

If applicable, is the statistical analysis and its interpretation appropriate?

Partly

Are all the source data underlying the results available to ensure full reproducibility?

Yes

Is the study design appropriate and is the work technically sound?

Partly

Are the conclusions drawn adequately supported by the results?

Partly

Are sufficient details of methods and analysis provided to allow replication by others?

Partly

Reviewer Expertise:

Cluster analysis; phenotype discovery; airways disease; health informatics.

We confirm that we have read this submission and believe that we have an appropriate level of expertise to confirm that it is of an acceptable scientific standard.

# Peer review: Reviewer response for version 2

**DOI:** 10.21956/wellcomeopenres.17575.r38965

**Published:** 2020-06-10

**Authors:** Maria Pikoula, Nonie Alexander

**Affiliations:** 1 Institute of Health Informatics, University College London, London, UK

The authors have responded in detail to our review and we welcome the changes they have made. We only have two further points to make, both relating to the section discussing cluster number selection. Addressing the first point should be straightforward and is of lesser importance. Addressing the second point is in our opinion essential, as the selection criteria for the model parameters (in this instance k) should be clearly stated.

1. (minor point) Ideally, the method of selecting the number of k should be presented in the methodology section and the findings (of the elbow plot and silhouette scores) should be presented in the results section.
2. (major point) It still remains unclear what the "visual inspection of maps" entails with regards to how k was selected. Is this, for example, purely geographical or geopolitical? Or was the similarity of countries assessed on the basis of the input variables, or perhaps the outcomes? The elbow plot and silhouette score both point towards the k=4 solution. Given that cluster analysis is generally used to uncover "hidden" patterns in data, then perhaps "dissimilar countries" were grouped due to some unmeasured factor(s). If, however the k=4 solution showed no "interesting" segmentation with regards to the outcome variables then this should be stated as it is sensible to reject it on that basis.

Is the work clearly and accurately presented and does it cite the current literature?

Yes

If applicable, is the statistical analysis and its interpretation appropriate?

Partly

Are all the source data underlying the results available to ensure full reproducibility?

Yes

Is the study design appropriate and is the work technically sound?

Partly

Are the conclusions drawn adequately supported by the results?

Partly

Are sufficient details of methods and analysis provided to allow replication by others?

Partly

Reviewer Expertise:

Cluster analysis; phenotype discovery; airways disease; health informatics.

We confirm that we have read this submission and believe that we have an appropriate level of expertise to confirm that it is of an acceptable scientific standard, however we have significant reservations, as outlined above.

# Response

**Published:** 2020-06-10

**Authors:** Rodrigo M Carrillo-Larco

**Affiliations:** Imperial College London, UK

**Q1. The authors have responded in detail to our review and we welcome the changes they have made. We only have two further points to make, both relating to the section discussing cluster number selection. Addressing the first point should be straightforward and is of lesser importance. Addressing the second point is in our opinion essential, as the selection criteria for the model parameters (in this instance k) should be clearly stated.**

A1. We thank the reviewer for the much relevant comments.

**Q2. (minor point) Ideally, the method of selecting the number of k should be presented in the methodology section and the findings (of the elbow plot and silhouette scores) should be presented in the results section.**

A2. If the editors allow, we would rather keep the manuscript as is. We agree that the findings of the cluster selection process may be shown in the results section. However, we focused this as an epidemiological work, that took advantage of a solid machine learning methodology. In that line, the results section shows the clusters, countries and their profiles, i.e., epidemiological evidence. All aspects of the machine learning analytical process were included in the methods section.

**Q3. (major point) It still remains unclear what the "visual inspection of maps" entails with regards to how k was selected. Is this, for example, purely geographical or geopolitical? Or was the similarity of countries assessed on the basis of the input variables, or perhaps the outcomes? The elbow plot and silhouette score both point towards the k=4 solution. Given that cluster analysis is generally used to uncover "hidden" patterns in data, then perhaps "dissimilar countries" were grouped due to some unmeasured factor(s). If, however the k=4 solution showed no "interesting" segmentation with regards to the outcome variables then this should be stated as it is sensible to reject it on that basis.**

A3. The last statement by the reviewer is a close representation of our process; however, we would not only use the word “interesting”, but also “reliable” or “expected”. We are sorry this did not come across in the last version. By “visual inspection” we meant that, based on general knowledge (geographical, geopolitical and epidemiological), 4 clusters grouped countries with little in common; in other words, based on prior knowledge, they did not have strong reasons to be together. It is not just that 4 clusters were uninteresting, but the configuration would not fully agree with prior belief; though 5 or 6 clusters would make more sense. As explained in our prior answer, we focused this more like an epidemiological work, thus we did not “blindly” follow the elbow or silhouette estimates, but tried to understand, based on prior knowledge, whether the clusters were sensible or expected. We have edited the methods section and included these lines: *As explained above, the visual inspection of maps suggested that 3 or 4 clusters did not provide a good classification. That is, countries with no strong similarities were clustered. Visual inspection of the maps was based on geopolitical, geographical and epidemiological knowledge, in general and regarding the input variables. A segmentation in 4 clusters did not reveal interesting, reliable or expected groups; in other words, based on background knowledge, countries expected to be together were not. A segmentation in 5 and 6 clusters provided sensible results in accordance with prior knowledge. Overall, our choice of 5 and 6 clusters was sensible, based on prior knowledge and still supported by the analysed metrics (dendrogram and Silhouette).*

# Peer review: Reviewer response for version 1

**DOI:** 10.21956/wellcomeopenres.17350.r38663

**Published:** 2020-05-27

**Authors:** Maria Pikoula, Nonie Alexander

**Affiliations:** 1 Institute of Health Informatics, University College London, London, UK

Carillo-Larco *et al*. used freely available data sources to perform a country-level cluster analysis of COVID-19 related variables. The resulting clusters were validated against outcomes related to mortality and confirmed cases. A statistically significant difference was observed between clusters with regards to number of confirmed cases. There was no correlation between cluster membership and mortality outcomes.

 The study design and results are, for the most part, clearly presented, and the article is well-written. However, information is lacking with regards to both methodological aspects as well as the presented findings of the study. Most importantly, it is not clear what question the study is trying to answer.

**Is the study design appropriate and is the work technically sound?**

 1) In terms of the appropriateness of the study, besides the lack of similar studies in the literature, no further justification is given as to why this study design was selected. If the purpose of the study is to allow for prediction of COVID-19 outcomes, a predictive model might have been more appropriate. The rationale for selecting cluster analysis is not sufficiently explained. Furthermore, the selection of input variables seems to be based on their availability rather than evidence from the literature that would make them suitable candidates for inclusion. The authors mention in the discussion that these variables are “relevant”, however this claim is not substantiated.

**Are sufficient details of methods and analysis provided to allow replication by others?**

 2) The paragraph explaining the selection of principal components should be re-written as it is ambiguous whether the retention of three PCA components was pre-specified or whether keeping 100% of the explained variance was the original target. It is my understanding that four variables were used as input in the PCA and the first three components were selected, and that the three together explain 100% of the variance. It makes no sense for solely the third component to explain 100% of the variance, especially given that the output of PCA lists components in descending order of % explained variance. 

 3) Related to the comment above, It is mentioned that “three components gave the most reliable clusters”. By which metric was reliability assessed? If this is to do with cluster stability, typically this entails re-sampling the data and verifying cluster stability with regards to the cluster characteristics using a metric such as the Jaccard coefficient ^1^.

 4) The following sentence in the section labelled k-means needs rephrasing: “Regarding the number of clusters that optimises the function convergence to the centroids, we estimated a cost function which supported the choose of five and six clusters”. At the moment it is not clear which cost-function is being referred to and what is meant by estimating a cost function. I suspect the authors are referring to the standard k-means cost function, the sum of squared distances from each point’s cluster centre.

 5) It is not clear how the choice of 5 or 6 clusters was made. According to the elbow plot in Figure 1, the elbow point is at 4 clusters. It is also unclear how the clustering results were used for the purpose of selecting k “based on visual inspection of maps and boxplots”. The maps in Figure 2 are fairly similar between the 5- and 6-cluster solutions and the boxplots in Figure 3 also suggest that clusters 0, 3 and 4 remain the same with some countries in clusters 1, 2 of the 5-cluster solution redistributed between them and with the additional cluster 5 in the 6-cluster solution.

 6) There are more reliable metrics to aid with cluster selection, including the silhouette coefficient ^2^, and the GAP statistic ^3^. The elbow plot is simply a heuristic. The authors should at least explain their choice of method.

**If applicable, is the statistical analysis and its interpretation appropriate?**

 7) Although appropriate, the statistical analysis lacks further interpretation. The usefulness of the model could be illustrated by evaluating the predictive value of cluster labels to answer the question “Are the labels more predictive than individual variables?” 

 8) The resulting clusters are difficult to interpret without a summary table of cluster characteristics in terms of the 4 input variables used in the analysis. 

**Are the conclusions drawn adequately supported by the results?**

 9) No specific conclusions are drawn in the discussion. What are the cluster characteristics and how are they associated with confirmed COVID-19 cases? Are the results expected, surprising? There is little discussion on the characteristics, whether present or absent in the model, that would drive the countries to cluster together with regards to the number of reported cases. A few example points for discussion are listed below.

 10) It appears from the map distribution that the clusters loosely correlate with GDP - although without a summary table confirming this is hard to tell for certain. I am not an epidemiologist and neither is NA, therefore it is not our area to comment, but countries with higher GDP are more likely to perform more tests, and are thus more likely to have a higher number of cases. 

 11) Additionally, some countries are more connected than others (e.g. because of air travel), and the spread of COVID-19 is not uniform across the world (e.g. countries that are closer to China reported cases earlier) and therefore, different countries are at different stages of the pandemic. It would make more sense to separately cluster countries with similar exposure to the virus as well as comparable reporting standards.

 Minor edits:

1. Figure 1 needs axis labels.

Is the work clearly and accurately presented and does it cite the current literature?

Yes

If applicable, is the statistical analysis and its interpretation appropriate?

Partly

Are all the source data underlying the results available to ensure full reproducibility?

Yes

Is the study design appropriate and is the work technically sound?

Partly

Are the conclusions drawn adequately supported by the results?

Partly

Are sufficient details of methods and analysis provided to allow replication by others?

Partly

Reviewer Expertise:

Cluster analysis; phenotype discovery; airways disease; health informatics.

We confirm that we have read this submission and believe that we have an appropriate level of expertise to confirm that it is of an acceptable scientific standard, however we have significant reservations, as outlined above.

## References

[1] : Silhouettes: A graphical aid to the interpretation and validation of cluster analysis. *Journal of Computational and Applied Mathematics*.1987;20: 10.1016/0377-0427(87)90125-7 53-65 10.1016/0377-0427(87)90125-7

[2] : Comparing sets of patterns with the Jaccard index. *Australasian Journal of Information Systems*.2018;22: 10.3127/ajis.v22i0.1538 10.3127/ajis.v22i0.1538

[3] : Estimating the number of clusters in a data set via the gap statistic. *Journal of the Royal Statistical Society: Series B (Statistical Methodology)*.2001;63(2) : 10.1111/1467-9868.00293 411-423 10.1111/1467-9868.00293

# Response

**Published:** 2020-05-29

**Authors:** Rodrigo M Carrillo-Larco

**Affiliations:** Imperial College London, UK

**Reviewer #2**

**Q1. The study design and results are, for the most part, clearly presented, and the article is well-written. However, information is lacking with regards to both methodological aspects as well as the presented findings of the study. Most importantly, it is not clear what question the study is trying to answer.**

A1. We appreciate the comprehensive evaluation; the comments will most certainly improve our work. We have included more details about the methodology (please refer to answers 4, 5 and 6); moreover, we have further elaborated on the results and discussion (please refer to answers 7, 8, 9, 10 and 11).

More than pursuing a specific research question, we aimed to develop a classification model that, benefiting from simple and available ecological variables, could cluster countries according to COVID-related outcomes (number of cases and deaths). If anything, our research question would be: *can country characteristics before the COVID-19 pandemic be useful to cluster countries according to COVID-19 number of cases and deaths? *We have modified the last paragraph of the introduction to include this question.

**Q2. In terms of the appropriateness of the study, besides the lack of similar studies in the literature, no further justification is given as to why this study design was selected. If the purpose of the study is to allow for prediction of COVID-19 outcomes, a predictive model might have been more appropriate. The rationale for selecting cluster analysis is not sufficiently explained. Furthermore, the selection of input variables seems to be based on their availability rather than evidence from the literature that would make them suitable candidates for inclusion. The authors mention in the discussion that these variables are “relevant”, however this claim is not substantiated.**

A2. We agree that lack of evidence is not a strong justification, and we acknowledge we were not clear on our motivations. These have been further elaborated in the last paragraph of the introduction; these lines read: *Therefore, classification algorithms can reveal patterns to identify countries where the pandemic may have a similar effect. Countries could use this information to prevent worse-case scenarios given the cluster to which they belong. Global and regional organizations could use country clusters to organize similar aid to countries in the same cluster while prioritizing clusters likely to experience the worse outcomes.*

We certainly included variables that were readily available. However, we also chose variables that were closely related to the COVID-19 pandemic. The rationale behind our variable selection was explained in the paragraph immediately before the “Data analysis–clustering” sub-heading. In these lines, we elaborated on why we chose the selected variables, what their relationship may be with COVID-19, and why we did not choose other variables that could have been available as well. References were included to support our statements.

**Q3. The paragraph explaining the selection of principal components should be re-written as it is ambiguous whether the retention of three PCA components was pre-specified or whether keeping 100% of the explained variance was the original target. It is my understanding that four variables were used as input in the PCA and the first three components were selected, and that the three together explain 100% of the variance. It makes no sense for solely the third component to explain 100% of the variance, especially given that the output of PCA lists components in descending order of % explained variance.**

A3. We apologise for the misunderstanding, as it was the consequence of a miscommunication. A priori, we decided on three PCA variables. We included eight input variables (please refer to answer 8) and applied the PCA. As you inferred correctly, these three PCA variables retained or explained 100% of the variance. As you correctly pinpointed, it made no sense for solely the third component to explain 100% of the variance. We have modified the text in the “PCA” sub-heading to better reflect this procedure: *In this work, and to avoid losing information explained by the original eight predictors, we prespecified three PCA components; the three PCA components retained a variance of 1. This method of obtaining 100% as an explained variance imply keeping 100% of the information explained by the original eight predictors.*

**Q4. The following sentence in the section labelled k-means needs rephrasing: “Regarding the number of clusters that optimises the function convergence to the centroids, we estimated a cost function which supported the choose of five and six clusters”. At the moment it is not clear which cost-function is being referred to and what is meant by estimating a cost function. I suspect the authors are referring to the standard k-means cost function, the sum of squared distances from each point’s cluster centre.**

A4. We referred to the “elbow” plot (Figure 1). We have rephrased this sentence to make it clearer, that we were talking about the “elbow” plot in figure 1. Please, refer to answers 5 and 6 for details about other modifications made regarding the analysis and cluster selection.

  

**Q5. It is not clear how the choice of 5 or 6 clusters was made. According to the elbow plot in Figure 1, the elbow point is at 4 clusters. It is also unclear how the clustering results were used for the purpose of selecting k “based on visual inspection of maps and boxplots”. The maps in Figure 2 are fairly similar between the 5- and 6-cluster solutions and the boxplots in Figure 3 also suggest that clusters 0, 3 and 4 remain the same with some countries in clusters 1, 2 of the 5-cluster solution redistributed between them and with the additional cluster 5 in the 6-cluster solution.**

A5. Selection of 5 and 6 clusters was informed, mostly, by epidemiological knowledge about the countries, and how these were clustered. We did not choose 4 clusters, as the elbow plot would have suggested, because some countries were clustered with others they have little in common, epidemiologically speaking. This is what we meant by “visual inspection of maps and boxplots”. Mostly maps, though we also checked the boxplots. We have included a few lines the methodology section (“K-means” sub-heading) to explain our rationale: *…That is, five and six cluster classified countries in groups with shared socio-demographic and epidemiological profiles. Although five and six clusters provided similar groups, six clusters classified central Africa with greater detail, which could be useful for these countries and regional organizations. Overall, the function cost (elbow plot, Figure 1), paired with the overall results (boxplots and maps), suggested that five or six clusters were a sensitive decision.*

The maps with 5 or 6 clusters look similar. However, the map with 6 clusters classified countries in central Africa with greater detail. Although in the same sub-region, socio-economic and epidemiological differences provide unique features to these countries, that a 6-cluster model can identify. We have also included this argument in the new lines (please, refer to the text in italic in the previous paragraph).

Please, for further arguments about the choice of 5 and 6 clusters, referrer to answer 6.

  

**Q6. There are more reliable metrics to aid with cluster selection, including the silhouette coefficient ^2^, and the GAP statistic ^3^. The elbow plot is simply a heuristic. The authors should at least explain their choice of method.**

A6. We did not follow any of these methods because of the limited number of observations available; that is, the number of countries (analysis units) studied. Given the reduced number of observations, the elbow function would be fairly similar for the number of clusters close to the “elbow”. At this stage, it is advisable to subjectively assess which clusters gives the best information or correlates better with expert knowledge,[1] ^,^[2] rather than relying only on performance metrics. As requested, we have further elaborated on the rationale for the choice of method: *When there is a limited number of observations, as it is arguably in this analysis, the number of clusters around the “elbow” function (Figure 1) provides similar information. At this point, it may be advisable to select the number of clusters which relates better to expert knowledge. Therefore, we used visual inspection of maps and plots to decide on the number of clusters that provide the best results, grouping countries in consistent clusters with a similar background.*

In addition, to further elaborate on our current choice of method, for clarity, transparency and consistency, we have conducted further analysis. First, the dendrogram with Euclidean distances showed the 5 clusters was the optimum number; this agrees with our current choice. The Silhouette analysis showed the metrics summarised in the table below. These show that the largest metrics (>40%) were retrieved for 3, 4, 5 and 6 clusters (please, see rows highlighted in green). After visual inspection of the maps with 3 and 4 clusters, we agreed that these did not classify or stratify countries well. In order words, there were countries in one cluster that may not have strong similarities (at least in epidemiological or socio-demographic terms). Consequently, 5 and 6 clusters appeared to be better options; again, the average silhouette score agreed with our original choice.

**Number of clusters =** **Average silhouette score**

2 = 0.388107

3 = 0.433095

4 = 0.477838

5 = 0.444210

6 = 0.415063

7 = 0.382376

8 = 0.354897

9 = 0.362776

10 = 0.365564

We have included the following paragraph in the “K-means” sub-heading: *Post-hoc analysis suggested we made a sensible choice when selecting 5 and 6 clusters. A dendrogram with Euclidean distances showed that 5 clusters were the optimum number. Similarly, the Silhouette analysis revealed the largest average Silhouette score for 3 (0.43), 4 (0.48), 5 (0.44), and 6 (0.42) clusters; all other options from 1 to 10 clusters were below 0.40. As explained above, the visual inspection of maps suggested that 3 or 4 clusters did not provide a good classification. That is, countries with no strong similarities were clustered. Overall, our choice of 5 and 6 clusters was supported by the analysed metrics (dendrogram and Silhouette).*

**Q7. Although appropriate, the statistical analysis lacks further interpretation. The usefulness of the model could be illustrated by evaluating the predictive value of cluster labels to answer the question “Are the labels more predictive than individual variables?”**

A7. We have further discussed (interpreted) about the relationship between the input variables, the cluster configuration, and how these relate to the outcomes. Please, refer to answers 9 and 10 for further details on the new text.

Although interesting, the proposed research question is beyond the aims of this work. The research question and justification have been further elaborated (please refer to answers 1 and 2). Arguably, any cluster may predict better than individual variables. That is a strong argument in favour or risk prediction models, above and beyond risk/prognostic factors alone.

**Q8. The resulting clusters are difficult to interpret without a summary table of cluster characteristics in terms of the 4 input variables used in the analysis.**

A8. We have included a table showing the median and interquartile range of the eight input variables across clusters (Table 1).

There were eight input variables (Table 1); disease prevalence included 4 diseases. That is, four prevalence estimates hence the four variables in addition to air quality, GDP, universal health coverage index and proportion of male subjects in the country (eight input variables in total). We have included the following lines under the “Data sources” sub-heading to avoid confusions: … *that is, we used eight input variables for the cluster analysis: four diseases, air quality, gross domestic product per-capita, an universal health coverage index and the proportion of men in the country (Table 1).*

  

**Q9. No specific conclusions are drawn in the discussion. What are the cluster characteristics and how are they associated with confirmed COVID-19 cases? Are the results expected, surprising? There is little discussion on the characteristics, whether present or absent in the model, that would drive the countries to cluster together with regards to the number of reported cases. A few example points for discussion are listed below.**

A9. We have further discussed on the cluster characteristics (input variables) and how these may explain the clusters configuration in relation to COVID-19 outcomes. These lines in the discussion section read (“Results in context” sub-heading): *The input variables could potentially explain the clusters configuration. For example, cluster number four had the largest number of confirmed cases. This cluster also had the best universal health coverage index. It could be argued that such a strong health system is capable of performing tests to large populations, hence a large number of diagnosed cases. Conversely, cluster number two appeared to have the worst death rates; this cluster also had the largest tuberculosis prevalence as well as the smallest gross domestic product per capita and universal health coverage index. These epidemiological –large burden tuberculosis – and socio-demographic profiles could explain the high death rates.*

  

**Q10. It appears from the map distribution that the clusters loosely correlate with GDP - although without a summary table confirming this is hard to tell for certain. I am not an epidemiologist and neither is NA, therefore it is not our area to comment, but countries with higher GDP are more likely to perform more tests, and are thus more likely to have a higher number of cases.**

A10. We have further discussed how GDP, as an input variable in the clusters configuration, may relate to how the clusters reveal COVID-19 outcomes. Please, refer to the previous answer for details about the new text.

  

**Q11. Additionally, some countries are more connected than others (e.g. because of air travel), and the spread of COVID-19 is not uniform across the world (e.g. countries that are closer to China reported cases earlier) and therefore, different countries are at different stages of the pandemic. It would make more sense to separately cluster countries with similar exposure to the virus as well as comparable reporting standards.**

A11. It would difficult to separately cluster countries with similar exposure to the virus; it would be more difficult to identify a threshold to define “similar exposure to the virus”. This approach will make the clustering more complex, which we tried to avoid by selecting variables readily available yet closely correlated to COVID-19 (please refer to answer 2). In this line, comparable reporting standards are not a static measure. Countries have improved their reporting standards at different paces and through different means during the pandemic. Finally, both the exposure to the virus and reporting standards are characteristics of the pandemic. However, our aim was to use pre-pandemic characteristics.

We have further discussed the relevance of flights or connections. Please, refer to the discussion section for the new text (“Results in context” sub-heading): *The cluster configuration herein presented did not seem to group countries closer to China, where the pandemic started. In other words, countries with the first imported cases did not cluster together. This could mean that the selected input variables do not correlate well with, for example, travel frequency or population movement from China to nearby countries. Alternatively, this unexpected finding could suggest that the selected input variables are more relevant than proximity or connections between countries.* 

**Q12. Figure 1 needs axis labels.**

A12. We are providing a new figure with axis labels.

[1] Murugan Anandarajan, Chelsey Hill, Thomas Nolan. Practical Text Analytics: Maximizing the Value of Text Data. Chapter 7.5.1.

[2] Chia-Hui Chang, Zhi-Kai Ding, Categorical data visualization and clustering using subjective factors, Data & Knowledge Engineering, Volume 53,

Issue 3, 2005, Pages 243-262, ISSN 0169-023X.

# Peer review: Reviewer response for version 1

**DOI:** 10.21956/wellcomeopenres.17350.r38301

**Published:** 2020-05-05

**Authors:** Alan E. Hubbard

**Affiliations:** 1 Division of Biostatistics, University of California, Berkeley, Berkeley, CA, USA

The main purpose of this article is to promote the use of clustering methods (k-means specifically) for aggregating regions into a smaller set of coherent clusters based on regional level data (non-Covid) that can be compared with Covid disease outcomes (case counts, deaths, order). The methodology is straightforward, reduces the dimension of the problem from a number of distinct regions to clusters of "like" regions and then if it also correlated with Covid outcomes, could be used to both simplify the presentation of the results and possibly provide insights as to differences in the evolution of the epidemic in different regions. There are other unsupervised methods one could use as well as supervised methods (e.g., classification and regression trees). The results of this clustering exercise depend on the relevance of the data used to cluster on the dynamics of Covid, and thus as the understanding evolves, there are other sources that should be considered (e.g., mobility data).

Is the work clearly and accurately presented and does it cite the current literature?

Partly

If applicable, is the statistical analysis and its interpretation appropriate?

Yes

Are all the source data underlying the results available to ensure full reproducibility?

Yes

Is the study design appropriate and is the work technically sound?

Yes

Are the conclusions drawn adequately supported by the results?

Yes

Are sufficient details of methods and analysis provided to allow replication by others?

Yes

Reviewer Expertise:

Machine learning, causal inference, epidemiology.

I confirm that I have read this submission and believe that I have an appropriate level of expertise to confirm that it is of an acceptable scientific standard.
